# Ethnobotanical insights on the management of plant pests and diseases by smallholder farmers in Mpumalanga Province of South Africa

**DOI:** 10.1186/s13002-024-00711-x

**Published:** 2024-07-31

**Authors:** Kutullo N. Shai, Simeon A. Materechera, Stephen O. Amoo, Adeyemi O. Aremu

**Affiliations:** 1 Indigenous Knowledge Systems Centre, Faculty of Natural and Agricultural Sciences, North-West University Mmabatho, Private Bag X2046, Mmabatho, 2790 South Africa; 2 Medicinal Plants and Industrial Crop Division, Agricultural Research Council - Vegetables, Industrial and Medicinal Plants, Private Bag X293, Pretoria, 0001 South Africa; 3 Unit for Environmental Sciences and Management, Faculty of Natural and Agricultural Sciences, North-West University, Private Bag X1290, Potchefstroom, 2531 South Africa

**Keywords:** Agrochemicals, Biocontrol, Biodiversity, Botanicals, Biotic stress, Crop protection, Food security, Indigenous knowledge, Sustainability

## Abstract

**Background:**

Pests and diseases are a major contributor to yield losses in sub-Saharan Africa, prompting smallholder farmers to seek cost-effective, accessible and ecologically friendly alternatives for crop protection. This study explored the management of pests and diseases affecting crops across eight selected villages in Ehlanzeni District, Mpumalanga Province, South Africa.

**Methods:**

A total of 120 smallholder farmers were purposefully selected utilising the snowball technique. Information on the management of plant pests and diseases was collected through interviews and focus group discussions using semi-structured interview schedules. Ethnobotanical indices, including relative frequency of citation (RFC), use-value (UV) and informant consensus factor (Fic), were used to quantify and rank the plants used for crop protection in the study area.

**Results:**

Twenty-three plant species (16 naturalised exotics and seven indigenous plants) belonging to 16 families were used for managing pests (vertebrates and invertebrates) and diseases (fungal and bacterial related) affecting crops in the study area. The dominant (100%) crops cultivated by the participants were *Allium cepa* L., *Mangifera indica* L., *Solanum lycopersicum* L. and *Zea mays* L. The RFC value ranged from 0.08 to 0.83 and the three most popular plants for crop protection were *Capsium annuum* L. (0.83), *A*. *cepa* (0.63) and *Dichrostachys cinerea* (L.) Wight & Arn. (0.43). In terms of the UV, the five most promising plants used as biocontrol were *Tulbaghia violacea* (0.13), *A*. *cepa* (0.12), *C*. *annuum* L. (0.09), *Solanum campylacanthum* Hochst. Ex A.Rich*.*(0.09) and *Pinus pinaster* (0.08). Based on the Fic, four categories were established and dominated by fungal diseases (0.64). Furthermore, *T*. *violacea* and *A*. *cepa* were the most often mentioned plants used against fungal conditions. Other categories cited were bacterial diseases (0.3), invertebrate pests (0.11) and vertebrate pests (0.14), an indication that smallholder farmers had limited agreement or common knowledge about the plants used for their management. The preparation methods included maceration (38%), decoction (38%) and burning (24%). Foliar application (67%) and soil drenching (33%) were used for administering plant extracts during the management of crop pests and diseases.

**Conclusion:**

The study highlights the importance of botanicals and associated indigenous knowledge among smallholder farmers in Mpumalanga Province, South Africa. It is pertinent to explore the valorisation of these botanicals by generating empirical data on their biological efficacies and phytochemical profiles.

**Supplementary Information:**

The online version contains supplementary material available at 10.1186/s13002-024-00711-x.

## Background

The importance for a country to be food secure cannot be overemphasised [1]. Food security is crucial for achieving the United Nations Sustainable Development Goals (UN SDGs) such as no poverty (UN SDG no.1), zero hunger (UN SDG no.2), as well as good health and well-being (UN SDG no.3) [2]. In developing countries, many households rely heavily on the agricultural sector for their food, livelihoods and general well-being [3]. Particularly, rural households practise subsistence agriculture, which often involves crop and/or animal production [4, 5]. Existing data indicate that approximately 15.3% of South African families are engaged in agriculture [6]. As one of the Provinces known for agricultural activities in South Africa, there is a high diversity of crops (including food and cash crops) cultivated in Mpumalanga Province [7]. As a re-occuring challenge facing crop production, pests and diseases cause significant losses and pose a significant threat to food security [8]. Globally, approximately 600 insect species are considered pests, while pathogenic fungi are responsible for significant biotic stress in agriculture [1, 9]. In sub-Saharan Africa, smallholder farmers are faced with the challenges of pests and diseases, exacerbated by climate change, resulting in yield losses and increased input costs with consequential negative effects on sustainable crop production and food security [10, 11]. In Mpumalanga Province, the incidences of pest and disease outbreaks with major detrimental effects on crop yield and quality remain a concern. Some of the major diseases affecting crop production include maize lethal necrosis, late blight, wheat rust and bacterial blight. The common pests include fall armyworms, leafhoppers, ants, termites, ladybirds, parasitic wasps, red-billed quelea, locusts and whiteflies [12–14].

Due to several factors including the increased risks to human health, the environment and the development of resistance by pests and pathogens, the use of agrochemicals (e.g. pesticides, fungicides and insecticides) in the management of plant pests and diseases is receiving more concerns among consumers [13, 15, 16]. Furthermore, agrochemicals can be poisonous to non-target creatures and have detrimental impacts on biodiversity when used excessively or improperly [14]. Chronic human diseases have been linked to components of agrochemicals, either through consumption or exposure [13]. The majority of agrochemicals are not readily biodegradable, which means that they build up in the environment and contaminate soil, groundwater and the ozone layer [14].

These aforementioned challenges are actively driving the need to source alternative and environmentally friendly approaches for managing pests and diseases affecting crops [17–20]. Globally, the use of plants for protection against pests and diseases dates back to over 3000 years ago [16]. People used parts of plants and their extracts as repellents against insects and pathogens. In Southeast Asia, Latin America and Africa, smallholder farmers have widely adopted the indigenous knowledge and practices of using plants and their extracts for the control of pests and diseases [12, 21–25]. In sub-Saharan Africa, several plants including *Aloe ferox* Mill., *A*. *vera* (L.) Burm.f., *Alstonia boonei* De Wild, *Allium cepa* L., *A*. *sativum*, *Annona squamosa* L., *Azadirachta indica* A. Juss, *Bidens pilosa* L., *Capsicum annuum* L., *C*. *frutescens* L., *Eucalyptus camaldulensis* Dehnh., *Lantana camara* L., *Nicotiana glauca* Graham., *N*. *tabacum* L. and *Tulbaghia violacea* Harv. have been documented as biocontrol agents against pests and diseases among rural communities [15, 26–28]. Different phytochemicals and antioxidants found in medicinal plants are important for the preservation and protection of crops [12]. When used in place of synthetic agrochemicals, plant extracts were efficient in reducing postharvest diseases of horticultural crops and increasing their shelf life [12]. Due to the limited documentation and preservation of these valuable approaches and resources, increased research interest and awareness have been recorded in recent times [29–31].

Crop production is one of the agricultural activities in the Mpumalanga Province of South Africa [32]. As currently experienced in other parts of the world, agricultural activity among farming communities within the Province is declining due to climate change. The region is also exposed to various risks resulting from insufficient agricultural management services in many local municipalities in the Province [32]. The provision of sustainable and effective management solutions is crucial for smallholder farmers, who significantly contribute to meeting the increasing food demands and household food security [33]. This can be through the application of indigenous knowledge based on natural resources utilised in crop production including land preparation, ploughing, planting, weeding, pest control and harvesting, thereby increasing food production [34]. Plant-derived pesticides and fungicides are recommended as a sustainable alternative to synthetic ones, improving crop production efficiency, mitigating food crises and protecting consumer health. These eco-friendly, affordable and easily incorporated bio-controls are environmentally friendly. Using indigenous biocontrol techniques is a practical and environmentally beneficial choice [29]. To develop appropriate and cost-effective solutions, it is important to assess how smallholder farmers utilise botanicals and associated indigenous knowledge for the management of pests and diseases.

In the Mpumalanga Province of South Africa, the utilisation of botanicals and associated indigenous knowledge among smallholder farmers for the management of crop pests and diseases remain orally transmitted with limited documentation. The untapped potential associated with the systematic documentation and preservation of indigenous knowledge has prompted the need for ethnobotanical studies among communities with anecdotal evidence on the use of botanicals for the management of pests and diseases. This study was aimed at gaining ethnobotanical insights into the use of botanicals for the management of pests and diseases by smallholder farmers in the Mpumalanga Province of South Africa. The objectives of the study were:

- To document the common pests and diseases affecting crops grown by smallholder farmers in the study area;
- To record the plant species that are used by the smallholder farmers to control pests and diseases; and
- To investigate how the smallholder farmers prepare and use the plants to control pests and diseases

The guiding research questions are outlined below:

- What pests and diseases affect crops that are grown by smallholder farmers?
- Which plant species are used to control the identified pests and diseases?
- How do smallholder farmers use plants to control pests and diseases?

## Materials and methods

### Description of the study area

Ehlanzeni District Municipality is in the north-eastern part of the Mpumalanga Province. The district has a land mass of approximately 27 895 km^2^ and a population of 1,970,000 [35]. It is bordered on the south by Gert Sibande District, on the north by Mopani and Sekhukhune Districts (Limpopo Province) and the west by Nkangala District Municipality. Bushbuckridge, City of Mbombela, Nkomazi and Thaba Chweu are the four local municipalities that make up the district. Mpumalanga Province has about 68% of its land devoted for agriculture. Tourism, forestry and agriculture are the main economic sectors that shape the patterns of land use in Ehlanzeni District. Agriculture is one of the key sectors in the economy of Ehlanzeni District Municipality, and it had the highest positive growth rate in 2017, with an average growth rate of 18.3% [32]. The region is in the summer rainfall region with raining season occurring from October to March. The Ehlanzeni area has between 750 and 860 mm of precipitation on average each year (Additional File 1: Supplementary Table S1). Mpumalanga Province is well endowed with diverse valuable agricultural products [7]. Macadamia nuts, groundnuts, sugar cane, coffee, tea, cotton, tobacco and citrus are among the common crops produced in Ehlanzeni North and South of Mpumalanga Province [7].

**Fig. 1:**
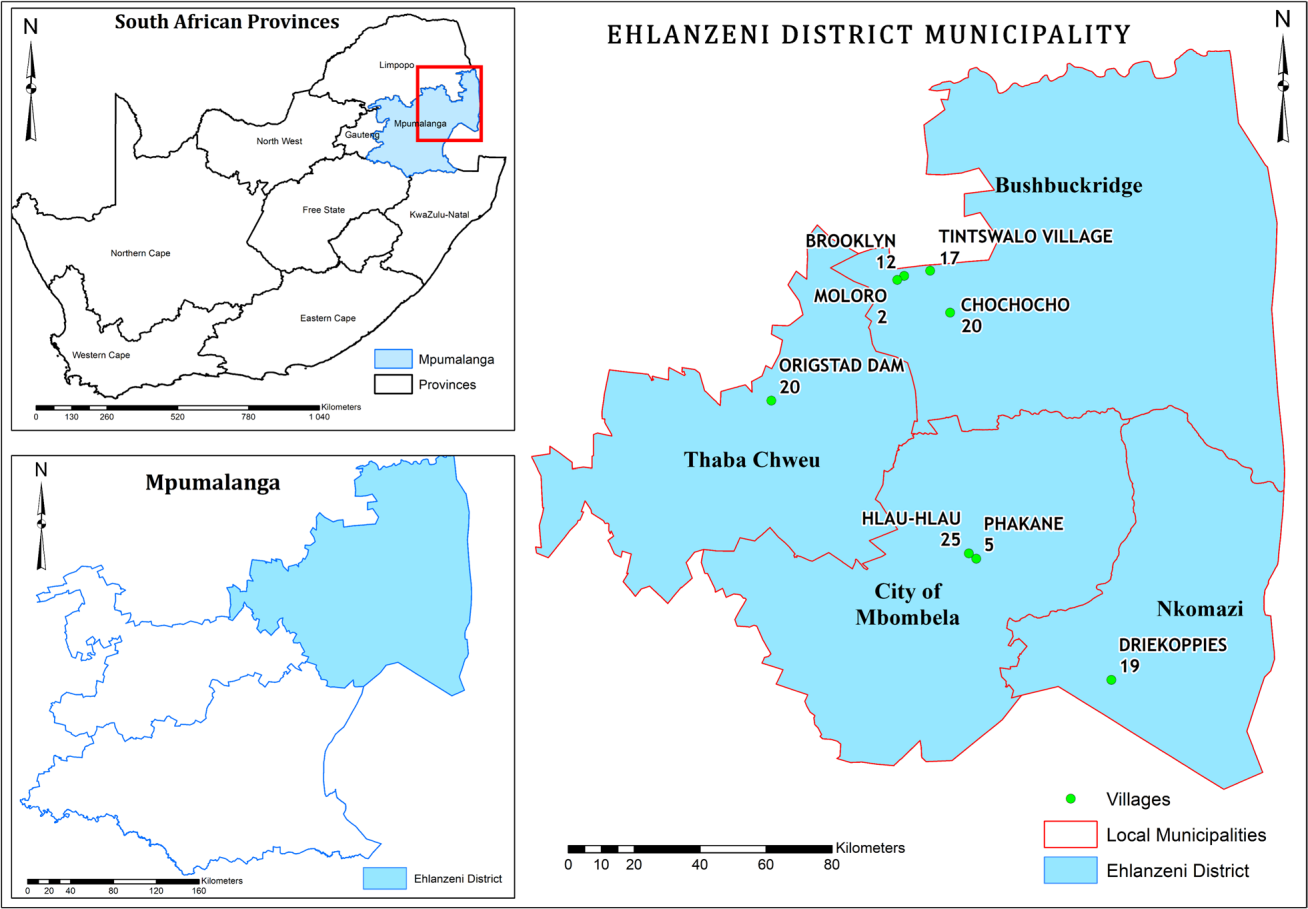
Overview of the selected villages that were studied in Mpumalanga Province, South Africa. The selected local municipalities of Bushbuckridge, Mbombela, Nkomazi and Thaba Chweu in Ehlanzeni District municipality

The study was conducted in eight villages, located within four local municipalities of Ehlanzeni District Municipality in the Mpumalanga Province of South Africa (Fig. 1, Additional File 1: Supplementary Table S1). The villages selected were Chochocho (24.7014 S, 31.1169 E), Brooklyn (24.3625 S, 30.5851 E), Moloro (GPS coordinates: 24.6078 S, 30.9787 E), Tintswalo village (24.3435.1408 S, 31.429.9064 E), Origstad dam (24.95357 S, 30.62978 E), Drikoppies (25.6991 S, 31.5638 E), Hlau Hlau (25.3566 S, 31.1764 E) and Phakane (25.3708 S, 31.1912 E) due to the presence of smallholder farmers in these areas (Table 1). Ehlanzeni District municipality is dominated by Blacks (94.15%), while the rest of the population groups consist of white (4.85%), coloured (0.73%) and Asian (0.27%) [35].

**Table 1:** Demographic characteristics of the participants in the selected villages for the study (n = 120)

Characteristic Parameter		Frequency	Percentage (%)
Local municipality	Village		
Bushbuckridge	Chochocho	20	17
	Brooklyn	12	10
	Moloro	2	2
	Tintswalo village	17	14
Thaba Chewu	Origstad dam	20	17
Inkomazi	Drikoppies	19	16
Mbombela	Hlau Hlau	25	21
	Phakane	5	4
Age range (Years)	18–30	6	5
	31–40	29	24
	41–50	19	16
	51–60	35	29
	61 and above	31	26
Gender	Male	52	43
	Female	68	57
Marital status	Living together	44	37
	Married	30	25
	Never married	16	13
	Widowed	18	15
	Separated	12	10
Formal educational level	No schooling	29	24
	Primary	27	23
	High school	44	37
	Tertiary	10	8
Farming experience	Below 3 years	4	3
	3–6 years	6	5
	7–10 years	19	16
	Above 10 years	91	76
Ethnicity	Pedi	56	47
	Sotho	1	1
	Swati	40	33
	Tsonga	23	19
Language	Sepedi	56	47
	Xitsonga	23	19
	siSwati	40	33
	Southern Sotho	1	1
Religion	Christianity	83	69
	Traditionalists	37	31
Occupation	Farming	120	100

### Target population, sampling procedure and sample size

The study purposively targeted smallholder farmers applying plants and associated indigenous knowledge to manage pests and diseases affecting crops on their farms. A snowball sampling technique was used to identify a sample size of 120 smallholder farmers. The study population consisted of both male and female smallholder farmers who were above the age of 18, understood the local languages spoken in the study area and incorporated indigenous knowledge practices in crop production. Smallholder farmers were the unit of analysis and the targeted population because they have a certain understanding of indigenous knowledge for crop production and protection. The members of the community and smallholder farmers assisted with the identification of the targeted population by referrals [36].

### Data collection technique

An ethnobotanical survey was conducted in Ehlanzeni between November 2022 and November 2023. The study used a semi-structured interview schedule with closed- and open-ended questions. In-depth face-to-face interviews with participants were carried out to collect quantitative data on the frequency and value of plant species and their applications. In addition, qualitative data were applied to establish the indigenous knowledge of crop farming (crop cultivation and the utilisation of plant species against pests and diseases) among the smallholder farmers. The information provided by the participants during the interviews was recorded on a voice recorder and transcribed into text. The data collection tools were validated by a pilot test before data collection. Member checking to verify interview data with participants during and after data collection and triangulation was used to improve the reliability and trustworthiness of the data. All interviews were conducted in the vernacular languages of Sepedi, siSwati and Xitsonga with the help of research assistants who also served as translators. Some of the aspects focussed were crops cultivated, pest and disease management methods for various crops, preparation and administration techniques, and application frequency. The awareness and adoption of botanical-based pest and disease management options among the smallholder farmers were included in the semi-structured interview schedule.

### Analysis of data

The data gathered on ethnobotanical knowledge on the use of botanicals by the participants for the management of pests and diseases were analysed using thematic content and ethnobotanical indices. The quantitative data on the frequency, value and administration of plant species were analysed using descriptive statistics [37]. The qualitative data were analysed using thematic content analysis method [38]. The data were transcribed after the interviews and checked for saturation and coherence. To find patterns and themes, the data from different individuals were compared to one another. Ethnobotanical indices, such as the relative frequency of citations (RFC) and use-value (UV), were calculated and used to ascertain the significance of the plants used for managing crop pests and diseases by smallholder farmers in the study area [39, 40]. The proportion of participants claiming the use of a particular plant species was estimated by relative frequency of citation (RFC) which was calculated as shown below:

1 $$RFC = \left( {Np / N} \right)$$

where Np is the number of citations for a particular plant species and N is the total number of participants in the study [30].

The UV which is an ethnobotanical index that shows the relative importance of plant species known locally based on the number of recorded uses for each plant species was calculated using the formula by Tardío and Pardo-de-Santayana [30] as shown below:

2 $${\text{UV}} = {\text{ Ui}}/{\text{n}}$$

where Ui is the total number of uses per species while “n” is the number of participants who reported on the plant species.

To determine the level of consistency among the participants on the knowledge regarding the use of plants for the management of pests and diseases affecting crops [41], the informant consensus factor (Fic) was calculated using the formula by Trotter et al. [41] as shown below:

3 $$Fic = \frac{{{\text{Nur}} - {\text{Nt}}}}{{\left( {{\text{Nur}} - 1} \right)}}$$

where Nur = number of used reports by participants for a particular plant-use category; Nt = number of taxa or species used for that use category for all participants; Fic values ranged from 0 to 1, where ‘1’ indicates the highest level of consent among the participants [41].

### Collection of herbarium samples for botanical identification and verification of plant species

Voucher specimens of all plants mentioned during the interviews were collected, prepared and deposited at the A.P. Goossens and S.D. Phalatse Herbariums, North-University, South Africa. The scientific names for the plant were verified using the ‘World Flora Online’.

(http://www.worldfloraonline.org, accessed on: 25 June 2024) and ‘Plants of the World Online’ (http://www.plantsoftheworldonline.org/, accessed on: 25 June 2024) [42].

### Ethical considerations

An ethics approval (NWU-01243–22-A9) was obtained from the Faculty of Natural and Agricultural Sciences Research Ethics Committee (FNASREC) of the North-West University, South Africa. A plant collection permit was issued by the Mpumalanga Provincial Department of Agriculture, Rural Development, Land and Environmental Affairs. To access the study areas, a letter of goodwill was issued by the Mpumalanga Provincial Department of Cooperate Governance and Traditional Affairs, and the Mpumalanga Provincial Department of Agriculture, Rural Development, Land and Environmental Affairs. Prior informed consent was obtained from all participants and consent forms were issued for signing before commencing the interview sessions. The consent form explicitly described the focus of the study and made it clear that participation in the data-gathering process was voluntary and that their personal information would not be shared without their agreement. To ensure confidentiality and protect the recorded information and interview schedule materials, all the participants were identified using codes. Appropriate socio and bio-cultural protocols were followed during the implementation of the study. Before the actual research, a pilot study was conducted at Thaba Sione village, Tswaing local municipality, Ngaka Modiri Molema District, North-West Province, South Africa. The use of photos and audio recordings taken during the research procedure helped to establish the reliability and trustworthiness of the study.

## Results and discussion

### Demographic characteristics of the participants in the study

An overview of the demographic characteristics of the participants is shown in Table 1. Most participants had completed their high school education. The Pedi ethnic group made up the majority of the participants who were interviewed in the study. Christianity was the most common religion in the research area. Sepedi is the most spoken language among the participants from the selected villages in the study area. The majority (57%) of the participants were females, reflecting the cultural roles of women in African communities when it comes to farming for household food security [43]. Consequently, women tend to possess more botanical knowledge and are skilled at gathering, preparing and using plants for different applications [43]. Women are often considered more knowledgeable in the use of indigenous knowledge for crop protection due to their involvement in diverse agricultural activities such as production, harvesting, storage, processing and marketing [31, 43–46]. On the other hand, males were the most dominating participants in the study conducted in the Eastern Cape Province [18]. Similar patterns were evident in other studies [28, 29, 44, 47], whereby males were the majority of the population that participated in farming practices involving the use of biocontrol for the management of crop pests and diseases. In the current study, participants in the age groups above 30 years (older generation) were more knowledgeable on plants and associated indigenous knowledge for crop protection than the younger generation (18–30 years). This may imply that young farmers tend to adopt modern methods learnt from schools rather than the traditional methods passed from generation to generation.

**Fig. 2:**
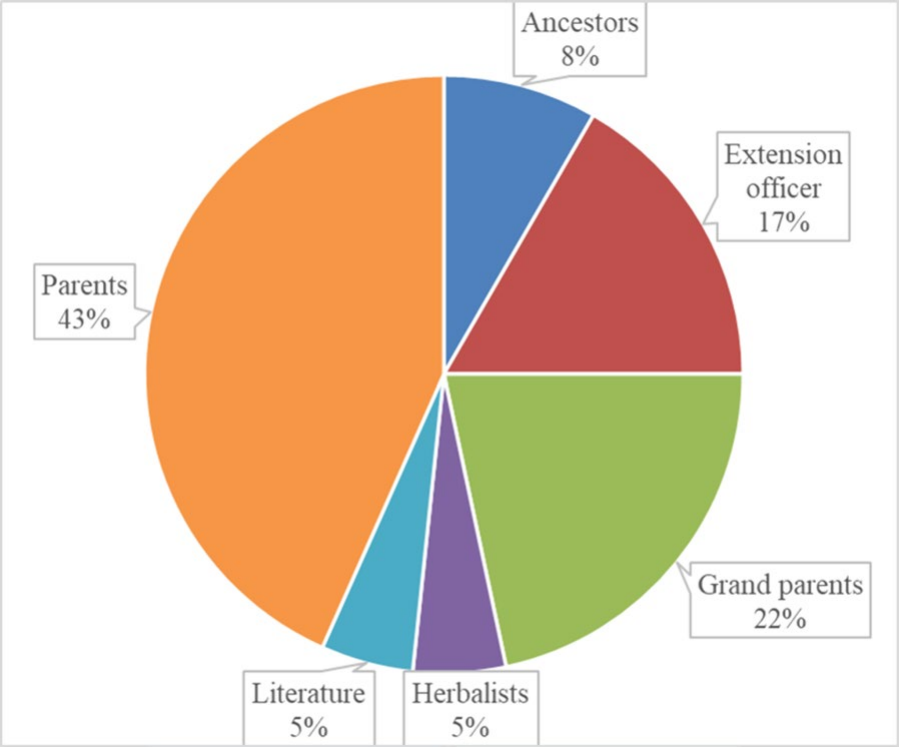
Sources of Indigenous knowledge of plants used for crop protection among the participants in Ehlanzeni District, Mpumalanga Province, South Africa (Number of participants, n = 120)

In this study, most (76%) of the participants had extensive (over 10 years) experience in farming **(**Table 1**)**. Similarly in the Eastern Cape Province, most smallholder farmers had more than 10 years of farming experience, practising traditional control of pests and diseases [18–20]. In the current study, the indigenous knowledge associated with the use of plants for crop protection was mainly inherited from parents (43%) and grandparents (22%) **(**Fig. 2**)**. Indigenous knowledge about plants for the preservation of crops is often hidden and regarded as sacred in many communities [48]. In the study area, all (100%) the 120 participants were self-employed.

### Land preparation, crops cultivated and local knowledge on common pests and diseases

In the Ehlanzeni District of Mpumalanga Province, the smallholder farmers cultivate their land per local customs and belief systems. The participants indicated that their traditional farming system was developed by the older farmers through generations following continuous interaction with the natural environment. Interestingly, the indigenous methods of cultivating crops without the need for outside assistance rely on locally accessible natural resources. Some of the indigenous strategies mentioned were field rotation to restore fertility and clearing fields by burning crop residues and biomass. Ploughing is applied in preparing the land for agricultural production and the participants use hand hoe “*Letsepe*” and work oxen “*Dikgomo*” to work the soil. Other land preparation methods included pegging, stumping and burning of grass. Harrowing was generally carried out after ploughing to break up larger soil clods and give a smoother surface for planting. In the study areas, weeds were managed during the growing season to prevent and minimise their spread and impacts on the crops. Most weeds were controlled by hand using a hoe or hand-pulling if the field was small. Other practices used to reduce weeds in the fields included mixed/intercropping and crop rotation. The participants emphasised the importance of maintaining soil fertility in fields used for cropping. Most farmers used organic fertilisers such as cow dung from the kraal, composted household and food waste, poultry manure and composted leaves from their gardens and farms.

The study revealed that the cultivation of crops was done all year round as different plants were grown at different times. This was done to ensure food security as the crops would mature at different times. The cultivation of crops was done by placing seeds when loosening and turning the soil, while the soil was still dry but when rainfall was anticipated. Indigenous knowledge and skills are frequently used by smallholder farmers to domesticate, enhance and preserve a variety of crops [34]. In total, 28 crops entailing five categories, namely forages, fruits, oils, vegetables, and tubers, were grown by the participants (Additional File 1: Supplementary Table S2). Solanaceae (four plant species), Amaranthaceae (three plant species) and Fabaceae (three plant species) were the families with the highest representation of the cultivated crops. The four crops with the highest frequency of citations (FC) with 100% mentions included onion, maize, tomato and mango. This was followed by chillies (94%), Jew’s mallow (83%), spinach (75%) and red amaranths (75%). The six crops with the lowest FC (1–8%) were apple (1%), cauliflower (1%), lettuce (2%), watermelon (4%), blackjack (8%) and prunes (8%). Out of the 28 cultivated crops, one plant species (Jew’s mallow) is indigenous while 27 (96%) are introduced/naturalised in terms of their biogeography. It was evident that the smallholder farmers mainly cultivate introduced or naturalised crops in their farmlands, which can also decrease biodiversity, compete with indigenous plant species for scarce resources and change habitats. The biological diversity of coexisting indigenous species may be severely impacted by invasive alien plant species, which can also degrade the quality of invaded habitats and potentially alter the way entire ecosystems function [49, 50].

The participants identified 15 crop pests including African striped skink (*Trachylepis striata*), ants (*Lasius niger*), aphids (Aphididae), armyworms (*Spodoptera frugiperda*), beetles (*Coleoptera* sp.), bugs (*Hexapoda* spp), cabbage looper (*Trichoplusia ni* (Hübner)), cutworms (*Agrotis ipsilon*), leaf miners (*Agromyzidae*), locusts (*Anacridium* spp*.*), snails (*Gastropoda*), termites (*Isoptera* spp), tree squirrel (*Sciurus*), rat (*Rattus norvegicus*) and root knot (*Meloidogyne arenaria*), as being prevalent in their farms **(**Tables 2 and 3**)**. The pests were classified into vertebrates (3) and invertebrates (12). The smallholder farmers reported that leaf miners (85%), aphids (75%), ants (67%), cutworms (67%), termites (56%) and armyworms (42%) were the major pests encountered. Similar pests including aphids caterpillars, spider mites and cutworms were also reported to be affecting crops in Kenya [51]. A total of 10 diseases caused by bacterial (2) or fungal (8) strains were identified as affecting crops in the study area. Black spot, brown blight, white rust and early blight were among the common fungal diseases affecting crops in the study area **(**Tables 2 and 3**)**. The two bacterial diseases reported were leaf spot (*Acidovorax konjaci*) and bacterial spot (*Vesicatoria* sp.) which generally had a low incidence of occurrence in the study area.

**Table 2:** Inventory of plants used for the management of pests and diseases affecting crops in villages of Ehlanzeni District, Mpumalanga Province, South Africa. The botanical names were confirmed and verified using the ‘World Flora Online’ (http://www.worldfloraonline.org/) and ‘Plants of the World Online’ (http://www.plantsoftheworldonline.org/). *Local/common name: S, Sepedi; Tso, Xitsonga; Swa, siSwati; Eng, English. UV = use-value. RFC = relative frequency of citation

Scientific name (Voucher number)	Plant family	Local/common names*	Preparation and administration	Targeted pest (s)	Targeted disease (s)	UV	RFC
*Acacia mearnsii* De Wild. (KNS 14)	Fabaceae	Black wattle (Eng); Mosheshet (S)	**Whole plant** is collected as firewood and burnt; the resultant ash is applied on the crops. Ash is mixed with 2-L water and drenched on the soil	Termites, ants, tree squirrel, cabbage looper, rat, African striped skink	Black spot	0.02	0.13
*Aloe arborescens* Mill. (KNS 22)	Asphodelaceae	Krantz aloe (Eng); Kgopa (S); Mangane (Tso)	**Leaves** are cut, macerated (soaked) in a 2-L water and sprayed on the crops	Termites, aphids, ants, and leaf miner, tree squirrel, rat, African striped skink, root knot	Black spot, root rot	0.05	0.22
*Allium cepa* L. (KNS 19)	Amaryllidaceae	Eie (S); Onion (Eng): Tinyala (Tso); Anyanisi (Swa)	**Bulbs** are carefully cut, macerated (soaked) in boiled water and sprayed on the crops	Armyworm, cutworm, aphids, ants, leaf miner, reptiles, bugs, snails	Damping rot, maize ear, black spot, downy mildew, powdery mildew, root rot, bacterial spot	0.12	0.63
*Allium sativum* L. (KNS 18)	Amaryllidacea**e**	Garlic (Eng); Kaliki (S); Igarlic (Swa)	**Bulbs** are carefully cut and macerated (soaked) in 5-L water bucket either cold or hot water and sprayed on the crops	Aphids, birds, armyworm, leaf miner	Black spot, bacterial spot	0.05	0.22
*Annona squamosa* L. (KNS 05)	Annonaceae	Motllepo wa sekgowa (S); Sugar apple (Eng)	**Whole plant** is collected as firewood and burnt; ash is applied on the crops. Ash is mixed with 2-L water and drenched on the soil	Termites, ants, tree squirrel, cabbage looper, rat, African striped skink	Black spot, bacterial spot, white rust	0.03	0.14
*Artemisia afra* Jacq. ex Willd. (KNS 15)	Asteraceae	Lenganangana (S); African wormwood (Eng)	**Whole plant** is carefully cut and macerated (soaked) with 5-L cold or boiled water and drenched on the soil	Aphids, snails, cutworm, leaf miner, termite	Unspecified	0.03	0.17
*Bidens pilosa* L (KNS 08)	Asteraceae	Blackjack (Eng) Moshitja (S)	**Whole plant** is macerated (soaked) and prepared as decoction (boiled) in 5-L water and sprayed on the crops	Aphids, armyworm, ants, tree squirrel, rat, African striped skink	Leaf spot, powdery mildew	0.04	0.08
*Capsicum annuum* L. (KNS 07)	Solanaceae	Chilli pepper (Eng); Pherefere (S); Virivirri (Tso); Epelepele (Swa)	**Fruit** is macerated (soaked) and prepared as decoction (boiled) in 5-L water and sprayed on the crops	Leaf miner, armyworm, ants, termites, aphids, cutworm, root knot, locust	Black spot, root rot, leaf spot, powdery mildew, white rust	0.09	0.83
*Carica papaya* L. (KNS 03)	Caricaceae	Mophopho (S); Papaya (Eng)	**Leaves** are macerated (soaked) in 5-L water and sprayed on the crops	Aphids, armyworm, ants, leaf miner, grasshopper, termites, cutworm	Damping rot	0.06	0.13
*Cannabis sativa* L. (KNS 10)	Cannabaceae	Patja (S); Mbange (Tso.)	**Leaves** are macerated (soaked) in 5-L water and sprayed on the crops	Aphids, armyworm, ants, tree squirrel, rat, African striped skink	Leaf spot, powdery mildew	0.04	0.17
*Cnidoscolus aconitifolius* (Mill.) I.M. Johnst*.* (KNS 02)	Euphorbiaceae	Chaya (Eng); Sepeneše sa chaya (S); Xipinichi xa chaya (Tso); Sipinachi se chaya (Swa)	**Leaves** are macerated (soaked) in 5-L water and sprayed on the crops	Armyworm, aphids, termites, cutworms	Leaf spot, powdery mildew	0.05	0.14
*Dichrostachys cinerea* (L.) Wight & Arn. (KNS 09)	Fabaceae	Sickle bush (Eng); Dzenge (Tso.); Moreke (S)	**Whole plant** is collected as firewood and burnt; the resultant ash is applied on the crops. Ash is mixed with 2-L water and drenched on the soil and sprayed on the crops	Ants, beetle, cutworm, termites, leaf miner	Damping rot, maize ear, white rust	0.06	0.43
*Eucalyptus diversicolor* F. Muell*.* (KNS 17)	Myrtaceae	Karri (Eng); Mogamose (S)	**Whole plant** is collected as firewood and burnt; the resultant ash is applied on the crops. Ash is mixed with 2-L water and drenched on the soil and sprayed on the crops	Cutworm, ants, aphids, armyworm	Damping rot	0.04	0.31
*Mangifera indica* L. (KNS 12)	Anacardiaceae	Mango (Eng); Menku (S); Mongose (Tso)	**Stem** is collected as firewood and burnt; the resultant ash is applied on the crops. Ash is mixed with 2-L water and drenched on the soil and sprayed on the crops	Armyworm, leaf miner, ants, root rot, birds	Black spot, Damping rot,	0.07	0.18
*Manihot esculenta* Crantz. (KNS 04)	Euphorbiaceae	Cassava (Eng); Morogo wa Motombhula (S); Ntsumbula (Tso)	**Leaves** are macerated (soaked) in 5-L water and sprayed on the crops	Aphids, cutworm, leaf miner, termites, armyworm	Black spot	0.07	0.21
*Moringa oleifera* Lam*.* (KNS 06)	Moringaceae	Moringa (Eng); Mohlare wa kotana ya moropa (S);	**Leaves** are macerated (soaked) in 5-L water and sprayed on the crops	Termites, cutworm, leaf miner, ants, armyworm, termites	Black spot	0.06	0.17
*Nicotiana tabacum* Vell. (KNS 13)	Solanaceae	Blara (S); Tobacco (Eng);	**Leaves** are macerated (soaked) in 5-L water and sprayed on the crops	Leaf miner, armyworm, ants, termites, aphids	Damping rot, maize ear	0.06	0.16
*Pinus pinaster* Aiton. (KNS 11)	Pinaceae	Mopai (S); Pine tree (Eng.)	**Whole plant** is collected as firewood and burnt; the resultant ash is applied on the crops. Ash is mixed with 2-L water and drenched on the soil and sprayed or applied on the crops	Aphids, cutworm, leaf miner, ants, armyworm, termites	Black spot, bacterial spot	0.08	0.13
*Sclerocarya birrea* (A. Rich.) Hochst. (KNS 01)	Anacardiaceae	Marula (Eng); Mohlare wa Morula (S); Nkanyi (Tso.); Sihlahla Sebuganu (Swa)	**Whole plant** is collected as firewood and burnt; the resultant ash is applied on the crops. Ash is mixed with 2-L water drenched on the soil and sprayed or applied on the crops	Cutworm, termites, leaf miner	Damping rot	0.03	0.17
*Solanum campylacanthum* Hochst. ex A.Rich*.*(KNS 16)	Solanaceae	Tholwana (S); Goat bitter-apple (Eng); Nduloane (Tso)	**Fruit** is macerated (soaked) and prepared as decoction (boiled) in 5-L water and sprayed on the crops	Termites, armyworm, leaf miner, ants, armyworm, aphids, cutworm, locust, tree squirrel, rat, African striped skink	Black spot, maize ear	0.09	0.21
*Strychnos madagascariensis* Poir (KNS 23)	Loganiaceae	Mogwagwa (S); Nkwakwa (Tso)	**Whole plant** is collected as firewood and burnt; the resultant ash is applied on the crops. Ash is mixed with 2-L water and drenched on the soil, sprayed or applied on the crops. Macerated (soaked) and sprayed on the crops	African striped skink, cutworm, bugs, root knot	Damping rot, leaf spot, powdery mildew	0.06	0.25
*Tulbaghia violacea* Harv. (KNS 21)	Amaryllidaceae	Wild garlic (Eng); Konofolo (S)	**Whole plant** is macerated (soaked) and as decoction (boiled) in 5-L water and sprayed on the crops	Aphids, birds, armyworm, leaf miner, termites, ants, cutworm, locust, root knot	Black spot, bacterial spot, damping rot, maize ear, black spot, downy mildew, powdery mildew	0.13	0.42
*Zingiber officinale* Roscoe. (KNS 20)	Zingiberaceae	Ginger (Eng); Gemere (S);	**Whole plant** is prepared as decoction (boiled) in 5-L water and sprayed on the crops	Aphids, root knot, cutworm, leaf miner, tree squirrel, rat, African striped skink	Black spot, root rot, bacterial spot	0.04	0.17

**Table 3:** Categorization for pests and diseases affecting crops in relation to the informant consensus factors of plant species used by smallholder farmers in Ehlanzeni District Municipality of Mpumalanga Province, South Africa. *Nur* denotes the number of usage reports for a certain disease category, whereas *Nt* denotes the variety of plants cited for the treatment of that specific ailment category, *Fic* = Informant consensus factor. Local name: S, Sepedi; Tso, Xitsonga; Swa, siSwati

Categories	Pests/diseases	Local name	Citation	Nur	Nt	Fic
Vertebrate pests				22	19	0.14
	Tree squirrel (*Sciurus*)	Sehlora sa sehlare (S); Maxijani (Tso); squirrel sesiHlahla (Swa)	7		6	
	Rat (*Rattus norvegicus*)	Legotlo (S); Kondlo (Tso); Ligundvwane (Swa)	7		6	
	African Striped Skink (*Trachylepis striata*)	Mokgaritswane wa methalo (S); Kolombyani (Tso); Umgololo lonemigca (Swa)	8		7	
Invertebrate pests				122	109	0.11
	Ants (*Lasius niger*)	Ditshosane (S); Vusokoti (Tso); Tintfutfwane (Swa)	15		16	
	Aphids (family: Aphididae)	Dintadimela (S); Tilwanyana letincane letimuya ijuusi (Swa)	16		16	
	Armyworms (*Spodoptera frugiperda*)	Mogokong (S); Ndzungula (Tso)	16		16	
	Beetles (*Coleoptera* sp)	Xitsotswana (Tso); ibhungane (Swa); Digogolaboloko	1		1	
	Bugs (*Hexapoda* Spp)	Podile (S); Xipembele (Tso)	2		2	
	Cabbage looper (*Trichoplusia ni* (Hübner))	Seboko sa khabetjhe (Tso)	2		2	
	Cutworms (*Agrotis ipsilon*)	Dibokosegi (S); Swivungu (Tso); Tibungu Letijutjiwe (Swa)	15		15	
	Leaf miners (*Agromyzidae*)	Matlakala a boepi (S)	15		15	
	Locusts (*Anacridium* spp)	Tšie (S)	4		4	
	Snails (*Gastropoda*)	Dikgopa (S); Tihumba (Tso); Iminenkhe (Swa)	2		2	
	Root-knot (*Meloidogyne arenaria*)	Lehuta la medu (S)	5		5	
	Termites (*Isoptera* spp)	Mohlwa (S); Majenje (Tso); Tilwanyana letincane etiphila ngemacembu lamakhulu (Swa)	15		15	
Bacterial diseases				11	8	0.3
	Bacterial spot (*Vesicatoria*)	Bokaaka bja mehlare ye twantsho (S); Emagciwane lamnyama emabala (Swa)	6		5	
	Leaf spot (*Acidovorax konjaci*)	Bahlasedi ba letlakala (S); Bahlaseli beMacembe (Swa)	5		3	
Fungal diseases				195	70	0.64
	Powdery mildew (*Podosphaera fusca* (Fr.) Braun & Shishkoff.)	Phoka ya leorle ya ka magareng (S)	7		7	
	Maize ear rot (*Gibberella zeae*)	Bahlasedi bahlogo ya mahea (S); Bahlaseli beNdlebe ye mmbila (Swa); Vahlaseri va tindleve tamvele (Tso)	5		2	
	Damping rot (*Pythium* spp. Pringsh)	Mavabyi ya kuthambat (Tso)	9		9	
	Downy mildew (*Peronospora sparsa* Berk)	Phoka ya ka fasana ya ka magareng (S)	4		4	
	Brown blight (*Colletotrichum lindemuthianum*)	Mavabyi ya brown blight (Tso)	50		15	
	Early blight (*Alternaria solani*)	Mavabyi ya early blight (Tso)	20		20	
	Black spot (*Diplocarpon rosae*)	Mavabyi ya black spots (Tso)	78		7	
	White rust (*Albugo candida*)	White ruse (S); I-ruse lehlomphe (Swa)	20		10	

### Application of Indigenous knowledge and use of plants for managing pests and diseases affecting crops

The current study revealed that using indigenous pest and disease management techniques is a practical and environmentally beneficial approach to the local communities. Given that Indigenous knowledge was still regarded as being crucial and as part of the heritage of the community, reliance on indigenous methods for managing pests and diseases was common and highly appreciated [48]. The smallholder farmers indicated that they apply both indigenous knowledge practices (80%) and modern approaches (20%) to control pests and diseases affecting their crops. The indigenous-based pest and disease management practices were prevalent and highly valued because they are considered relatively accessible and affordable within the communities. However, some participants raised a concern as the indigenous knowledge was mostly known by the elderly and is at risk of being eroded, particularly among the younger generation. The lack of documentation and access was the main barrier preventing indigenous knowledge from being used for managing crop pests and diseases by many participants within communities. Another risk expressed was that as elderly persons age and pass on, their indigenous knowledge on pest and disease management may be lost. The participants also indicated their attitudes on utilising indigenous knowledge had changed as a result of exposure to contemporary pest and disease management methods especially the use of synthetic chemicals. Van den Ban et al. [40] acknowledged that the knowledge of local farmers is essential to the development of sustainable agriculture because this method of farming is adapted to local conditions.

The wide adoption of environmentally friendly practices including the use of botanicals and associated indigenous knowledge have the potential to reduce the amount of harmful, non-biodegradable substances that end up in the environment, especially in the water bodies [29]. The participants also stated that they regularly monitor the field during crop growth to determine when weeds should be pulled out and to choose plant combinations that will allow other plants to act as pesticides or pest repellents due to their aroma. A few of the participants managed crop pests in the field and during storage by using smoke, scarecrows and traps. The use of plants in the management of crop pests and diseases was also prevalent in the current study **(**Table 2**)**. Researchers have explored the potential of plant extracts and essential oils from a variety of botanicals to protect crops against pests and diseases [12, 25, 52]. Different phytochemicals are effective for the preservation and protection of crops. Extracts from plants including *Zingiber officinale*, *A*. *vera*, *A*. *cepa* and *A*. *sativum* are efficient at reducing postharvest diseases of horticultural crops and extending their shelf life [12, 23, 24].

The current study recorded 23 plant species belonging to 16 families that were utilised by smallholder farmers to manage crop pests and diseases **(**Table 2**)**. The RFC for the identified plants varied from 0.08 to 0.83, which is a measure of their popularity among the participants. Furthermore, the top 10 cited plant species had RFC values that ranged from 0.21 to 0.83. These 10 most cited plants were *C*. *annuum*, *A*. *cepa*, *D*. *cinerea*, *T*. *violacea, E*. *diversicolor*, *S*. *madagascariensis*, *A*. *arborescens*, *A*. *sativum*, *S*. *campylacanthum* and* M*. *esculenta*. We recorded UV as a measure of the diverse uses that ranged from 0.02 to 0.13 for the 23 plant species in the study area. The top plant species exerting diverse uses were *T*. *violacea* (0.13), *A*. *cepa* (0.12), *C*. *annuum* (0.09), *S*. *campylacanthum* (0.09), *Pinus pinaster* (0.08), *M*. *esculenta* (0.07), *M*. *indica* (0.07), *D*. *cinerea* (0.06), *S*. *madagascariensis* (0.06), *Moringa oleifera* (0.06), *N*. *tabacum* (0.06), *Carica papaya* (0.06), *A*. *sativum* (0.05) and *A*. *arborescens* (0.05).

To identify plants that are culturally significant for crop management in the study area, the Fic ranged from 0.11 to 0.64 for the four categories of pests and diseases affecting crops (Table 3). The Fic values for different disease categories often depend on the availability of plants in the research region [53]. This study revealed that there is an agreement among the participants in the management of fungal diseases affecting crops, with the highest Fic of 0.64. Furthermore, the fungal diseases were often managed with some of the most cited and multipurpose plants such as *T*. *violacea* and *A*. *cepa*. Generally, high Fic values suggest that the knowledge about the plant species used for crop management are reasonably reliable [17–19, 54]. There was no consensus or shared knowledge among smallholder farmers regarding the plants used to manage pests and bacterial diseases affecting crops, as evidenced by the lower Fic values for vertebrate pests (0.14), invertebrate pests (0.11) and bacterial diseases (0.3).

Plant species such as *T*. *violacea*, *A*. *ferox*, *A*. *cepa*, *C*. *annuum* used to manage pests and diseases affecting spinach cabbage have been documented through surveys in the villages of South Africa [17–19, 54]. In Nigeria, similar plants (e.g. *N*. *tabacum*,* C*. *annuum*,* C*. *frutescens, Z*. *officinale, M*. *oleifera*) recorded in the current study were used to manage pests and diseases affecting crops [55–57]. The use of plant extracts against pests and diseases affecting crops has gained momentum due to their eco-friendly nature, availability and biodegradability [58]. They contribute to a reduction of the negative effects that pests and diseases have on humans, animals and the environment. Utilising plants may result in lower usage of synthetic pesticides, fewer outbreaks of pests and diseases, and better soil health. Generally, medicinal plants are locally available, cost-effective and environmentally friendly [18]. In addition, plants help to improve the soil as their chemicals may be released into the soil,  serving a defensive role against phytopathogens [59].

Solanaceae, Amaryllidaceae, Anacardiaceae, Asteraceae and Euphorbiaceae were the dominant families with the highest number of plant species used to manage crop pests and diseases. Particularly, families such as Solanaceae, Asteraceae and Amaryllidaceae have been reported to contain plant species used to manage crop diseases in the Eastern Cape Province of South Africa [18, 19]. In Nigeria, similar plant families such as Euphorbiaceae, Fabaceae, Amaryllidaceae and Solanaceae are utilised to manage insect pests affecting crops [55, 56]. In Uganda, Mwine et al. [44] reported families such as Asteraceae, Euphorbiaceae and Solanaceae for the management of pests and diseases. Plant families such as Asteraceae, Fabaceae and Solanaceae are highly valued for their therapeutic properties and were found to be among the most frequently used families in ethnopharmacology [46, 60] (Additional file 1).

#### Empirical evidence on the biological effects and phytochemicals for some of the recorded plants utilised for managing crop pests and diseases

High RFC for plant species could be indicative of their potential efficacy in managing agricultural pests and diseases. It could also be due to their relative accessibility and/or availability. Due to their accessibility and low cost, botanicals are widely used by farmers in developing nations to protect their crops [28]. The presence of plants used for crop protection could be suggestive of the extensive knowledge and dependence on botanicals for biocontrol among the selected communities. Medicinal plant extracts offer advantages in biocontrol due to their bioactive compounds, low environmental persistence and low cost, making them beneficial for smallholder farmers with limited resources [61, 62]. Medicinal plants produce secondary metabolites with antimicrobial properties, offering a potential non-toxic and cost-effective alternative to chemical fungicides [63, 64]. These compounds including terpenes and phenolics are known to inhibit the growth of microorganisms [45, 65]. Generally, the antimicrobial compounds in plants may be involved in plant defence against microbial pathogens [52]. Despite the high number of plants that have not been evaluated for their antimicrobial effects related to plant diseases [64, 65], only limited promising biological effects related to crop protection have been reported across the globe.

In India, Muthukumar et al. [24] demonstrated that the extracts from the leaves of *A. sativum* and *A*. *cepa* exhibited the highest degree of suppression against *Pythium aphanidermatum* mycelial growth (13.7 mm). Based on a study conducted in Slovenia [23], potential antibacterial effect of *A*. *arborescens* gel against *Bacillus cereus* was recorded following its potent growth-inhibitory qualities. In South Africa, an in vitro study by Olajuyigbe et al. [25] revealed the fungicidal activity of *A*. *mearnsii* against *Aspergillus niger* and *Aspergillus flavus*. Furthermore in Sri Lanka, methanolic extracts of *Z*. *officinal*e effectively inhibited many phytopathogens, including *Fusarium oxysporum*, *Rhizoctonia solani* and *Colletotrichum musae* [21]. In Nigeria, Fagbohun et al. [22] revealed the fungicidal activity of *Cnidoscolus aconitifolius* against *Aspergillus tamarri* and *Aspergillus niger*. In addition, the phytochemical screening of *C*. *aconitifolius* leaves indicated the presence of alkaloids, saponin, tannin, flavonoids and cardiac glycoside [22].

In the study by Sangeetha et al. [62], the crown-rot disease was significantly reduced by 86% following the dipping of banana fruit in *A*. *cepa* and *A*. *sativum* extracts. *M*. *oleifera* exerted antifungal activity that was higher than or equal to that of the commercially available fungicide ketoconazole. Arredondo-Valdés et al. [61] found that ethanol extracts of *M*. *oleifera* leaves had a strong inhibitory impact against *Agrobacterium tumefeciens*, *Clavibacter michiganensis* subsp. *michiganensis*, *Pseudomonas syringae* pv. tomato, *Ralstonia solanacearum* and *Xanthomonas axonopodis*. Based on their findings, *M*. *oleifera* was recommended as a powerful bio-bactericide. In addition, *N*. *tabacum*, *M*. *oleifera* and *Z*. *officinale* extracts have been reported to control maize weevil [12, 61, 62, 66]. To combat cabbage pests, some communities utilise extracts of *A*. *cepa*, *A*. *sativum*, *A*. *vera*, *B*. *pilosa*, *C. annuum*, *N*. *tabacum* and *S*. *giganteum* [27, 54].

### Plant parts used to manage crop pests and diseases

**Fig. 3:**
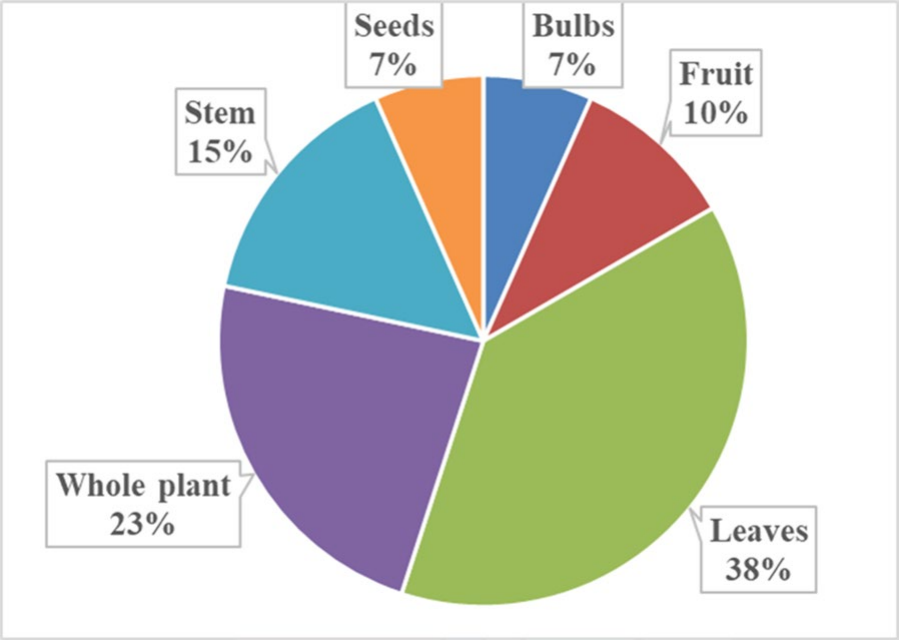
The plant parts that are used to manage pests and diseases affecting crops in Ehlanzeni District, Mpumalanga Province, South Africa (Frequency of plant parts, n = 60)

In this study, the main plant parts used for managing crop pests and diseases were leaves (38%), whole plant (23%), stem (15%), fruit (10%), and seed and bulbs at 7% **(**Fig. 3**)**. When compared to other plant parts, leaves are more readily available, and simpler to harvest and handle, which may also make them an attractive option for older farmers who form the majority when it comes to using botanicals [19]. Destructive harvesting of medicinal plants can lead to resource exhaustion and species extinction. Sustainable use of plants requires good harvesting practices [67, 68]. Other studies in Nigeria and South Africa have also reported the use of plant parts such as whole plants, leaves, stems, bark and bulbs for preparing pesticides from plants [17, 18]. In Eastern Cape Province, smallholder farmers often used leaves as the main plant part to manage crop pests and diseases [18, 19]. The ideal method for preserving biological resources and promoting sustainable bioprospecting, particularly for medicinal plants, will be to use leaves, which are more widely available than other plant parts. The study has indicated that participants used a conservation and harvesting strategy that involved regularly gathering plant leaves and shoots to extend the life of the plants.

### Mode of preparation and administration of plants used to manage crop pests and diseases

The plant extracts were prepared by the participants using various methods and formulations. The three extraction techniques for preparing the plants used were maceration (soaking in water), decoction (boiling in water) and burning (smoking). The dominant preparation techniques used for controlling crop pests and diseases were maceration and decoction (38%), followed by burning (24%). In terms of maceration, the participants indicated that they carefully soaked the dried and ground material in water in a 5-L container or bucket overnight and then used the formulation the next day. In the decoction method, participants indicated that they boiled the dried and ground material in water for 30 min and carefully placed it under shade to cool down before they applied it to the crops. The burning preparation method on the other hand entailed burning dried plant parts and allowing the smoke to repel the pests and insects on the farm or applying the burnt ash to the crops.

According to Anjarwalla et al. [69], different aspects, such as correct identification of the plant species, collection time, location, processing methods and application strategies, might affect pesticidal efficiency. Anjarwalla et al. [69] emphasised the importance of correctly identifying a plant and that the appropriate plant component is used, harvested at the appropriate time and extracted properly. In the current study, the participants indicated that before collecting and preparing the plants, they first observe and consult other smallholder farmers who have more knowledge than themselves. The participants indicated that they carefully and slowly dry the plant parts in the sun for days before preparing formulations and extracts from them.

Based on existing studies conducted in sub-Saharan Africa, smallholder farmers prepare and administer plant parts using a variety of methods and formulations to manage crop pests and diseases [19, 27, 33, 55, 70–74]. Some botanicals have strong biocontrol qualities, and they are frequently applied as repellents, fungicides, insecticides and rodent control agents. According to Chandola et al. [75], ash kills insects by either desiccating them or by filling the intergranular gaps, which prevents their movement or emerging during development.

In the study area, some plant species were grown/planted next to other crops, offering natural sources of smells that repel/bait insects from reaching the crops they intend to destroy. This was especially common among plant species such as marigolds and other fragrant flowering plants. In the study conducted by Skenjana et al. [20], four preparation techniques identified included boiling, mixing, soaking, as well as combining boiling and soaking. Although they might be more efficient than synthetic pesticides, formulations made of extracted parts from botanical plants should be explored for their ability to prevent disease damage and pest infestation on commonly produced crops in South Africa. It is crucial to be aware of the variables that could impact the effectiveness of botanicals, including differences in the amount of plant material and, thus, active chemicals, as well as variances in the formulation and preparation. As a result, the manufacturing and composition of these botanical pesticides need to be standardised.

**Fig. 4:**
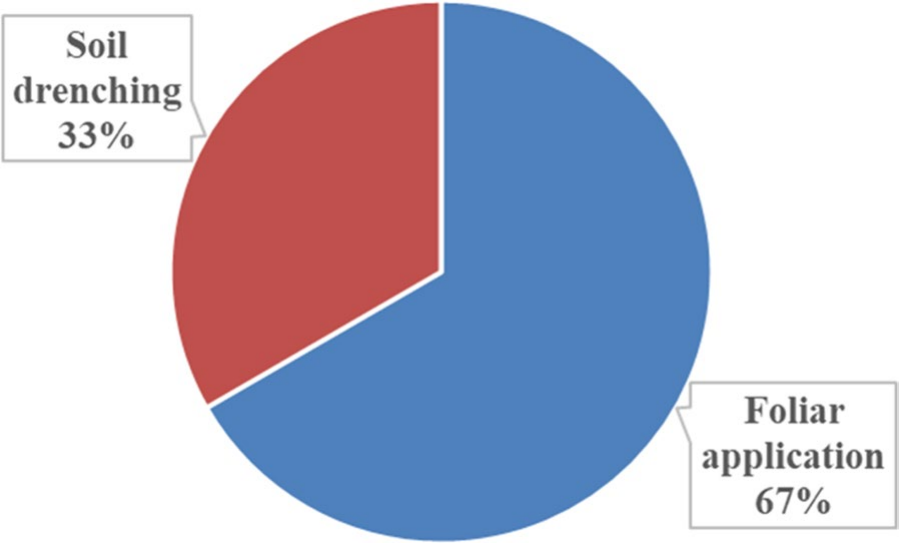
The application methods of the plant parts that are used to manage pests and diseases affecting crops in Ehlanzeni District, Mpumalanga Province, South Africa (Frequency of application methods, n = 69)

**Fig. 5:**
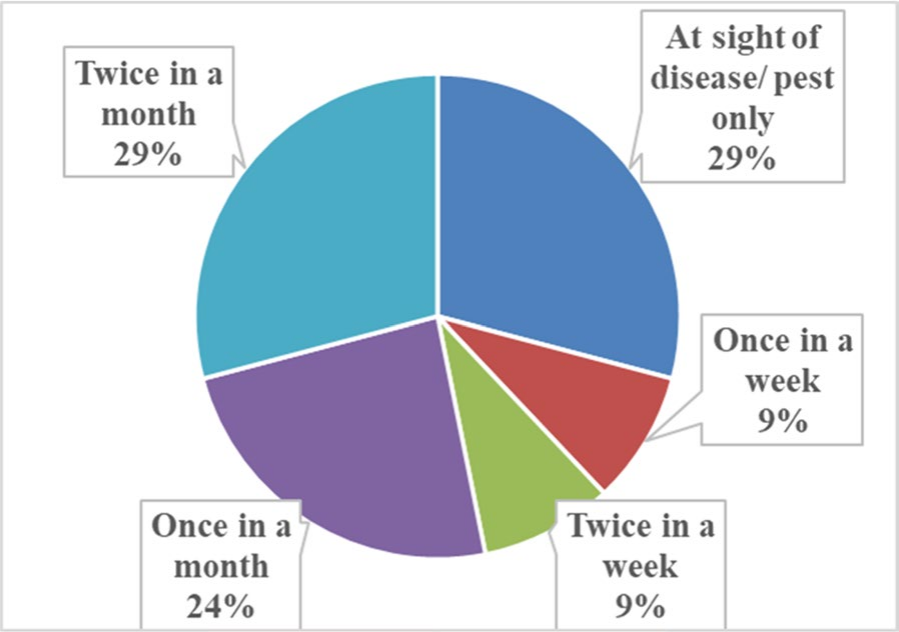
The application frequency of the plants that are used to manage pests and diseases affecting crops in Ehlanzeni District, Mpumalanga Province, South Africa (Frequency of application, n = 81)

The methods of administering the plant parts for managing crop pests and diseases included foliar application (67%) and soil drenching (33%) **(**Fig. 4**)**. In terms of spot treatment, dried ground material was applied on the spot of the crop leaves using 2-L bottles with holes made on the lid. The participants also explained that in terms of techniques used for soil drenching, they carefully opened the soil with a hoe and applied the plant material. The spreading and spraying of macerated, dried and boiled material were done with 2-L containers. Smallholder farmers use different procedures and formulations to prepare the plant extracts [27, 55, 57, 70]. For instance, decoction and maceration were the most often used methods for controlling agricultural pests and diseases in the Eastern Cape Province of South Africa [18]. In terms of application frequency, the methods were applied once a week and twice a week (9%), once a month (24%), at the sight of disease or pest infestation only and twice a month with 29% **(**Fig. 5**)**. Previous studies have indicated that common preparation techniques of plants used for managing pests and diseases in field crops include mixing, boiling and pasting [17–19, 54].

### Biogeography, conservation status and Indigenous strategies for plant species used for crop protection

In the study area, the 23 reported plants consisted of 16 naturalised exotics and seven indigenous species. For their continued existence, several indigenous communities have made biodiversity conservation a top concern [76, 77]. Out of the 23 recorded plants that are widely distributed in the Mpumalanga Province, seven of the reported plant species are classified as being of ‘least concern’ which indicates less threat to their survival in naturally occurring populations [78]. In terms of the recorded plant parts **(**Table 4**)**, harvesting the whole plant presents the greatest risk, followed by harvesting of bulbs and roots. Some of the participants indicated that they had noticed the limited availability of certain plants and expressed concern about their scarcity due to over-harvesting. Unsustainable harvesting methods may result in difficulty in finding valuable plant species including those used for crop protection against pests and diseases [69]. Given the general importance of plants for human and animal survival, as well as the need to meet the demands of both the present and future generations, their effective management, domestication, conservation and utilisation remain pertinent [12, 54].

**Table 4:** The reported availability status of plant species used by smallholder farmers to manage crop pests and diseases in Ehlanzeni District Municipality of Mpumalanga Province, South Africa

Plant species		Life form	^#^Biogeography status	Plant part used	Status as reported by the participants	*Conservation status
*Acacia mearnsii*		Tree	Naturalised exotic	Whole plant	Fairly abundant	Not evaluated
*Aloe arborescens*		Shrub	Indigenous	Leaves	Fairly abundant	Least concern
*Allium cepa*		Herb	Naturalised exotic	Bulb	Fairly abundant	Not available
*Allium sativum*		Herb	Naturalised exotic	Bulb	Fairly abundant	Not available
*Annona squamosa*		Tree	Naturalised exotic	Whole plant	Fairly abundant	Not available
*Artemisia afra*		Herb	Indigenous	Whole plant	Fairly abundant	Least concern
*Bidens pilosa*		Herb	Naturalised exotic	Whole plant	Fairly abundant	Not evaluated
*Capsicum annuum*		Herb	Naturalised exotic	Fruit	Fairly abundant	Not evaluated
*Carica papaya*		Tree	Naturalised exotic	Leaves	Fairly abundant	Not available
*Cannabis sativa*		Herb	Naturalised exotic	Leaves	Fairly abundant	Not evaluated
*Cnidoscolus aconitifolius*		Shrub	Naturalised exotic	Leaves	Rare	Not available
*Dichrostachys cinerea*		Shrub	Indigenous	Whole plant	Fairly abundant	Least concern
*Eucalyptus diversicolor*		Tree	Naturalised exotic	Whole plant	Fairly abundant	Not available
*Mangifera indica*		Tree	Naturalised exotic	Stem	Fairly abundant	Not evaluated
*Manihot esculenta*		Shrub	Naturalised exotic	Leaves	Fairly abundant	Not evaluated
*Moringa oleifera*		Tree	Naturalised exotic	Leaves	Rare	Not available
*Nicotiana tabacum*		Herb	Naturalised exotic	Leaves	Common	Not evaluated
*Pinus pinaster*		Tree	Naturalised exotic	Whole plant	Fairly abundant	Not available
*Sclerocarya birrea*		Tree	Indigenous	Whole plant	Fairly abundant	Least concern
*Solanum campylacanthum*		Herb	Indigenous	Fruit	Fairly abundant	Least concern
*Strychnos madagascariensis*		Tree	Indigenous	Whole plant	Fairly abundant	Least concern
*Tulbaghia violacea*	Herb		Indigenous	Bulb	Fairly abundant	Least concern
*Zingiber officinale*	Herb		Naturalised exotic	Bulb	Fairly abundant	Not available

^#^Biogeography status and *Conservation status were derived from the South African National Threatened Species Programme. SANBI: Red List of South African Plants [78]

Indigenous knowledge is used in the current study to conserve plant species and guarantee their survival for future generations. Traditional methods for preserving plant species include cultivating plants in backyards and only picking required plant parts including leaves, as they are easily accessible and regenerate. According to Skenjana et al. [19], leaves are readily available, cost-effective and easy to harvest, making them a popular choice for older farmers using botanical pesticides. In situ cultivation of plants in home gardens protects native species and preserves natural communities. Medicinal plant cultivation reduces wild population dependence, but overexploitation may lead to environmental degradation and genetic diversity loss [79, 80]. Other indigenous techniques and practices have been reported for the sustainable uses and conservation of plant resources. In some cases, people are discouraged from harvesting the entire plant but to harvest parts of the plant to allow for regrow and a continuous supply of materials in the future [81].

### Challenges faced by smallholder farmers in using indigenous knowledge systems in the management of crop pests and diseases

The study revealed that smallholder farmers face some challenges in adopting the use of indigenous knowledge systems for the management of crop pests and diseases. Due to the limited educational programmes, smallholder farmers are not aware of any frameworks for indigenous knowledge technologies in crop pest and disease management. Magocha et al. [34] reiterated that indigenous knowledge in crop production has been hindered by various obstacles. Regardless of the age or gender of a farmer, awareness and promotion of using indigenous knowledge systems in crop production and management are crucial. Additionally, the smallholder farmers were concerned about the frequent occurrence of migratory crop pests and diseases due to climate change. This prevented them from implementing proper indigenous management practices. Numerous studies have emphasised the significance of accessibility and awareness of indigenous knowledge on plant utilisation for their livelihoods [15, 17, 18].

## Conclusion

This study revealed that smallholder farmers in the Ehlanzeni District Municipality of Mpumalanga Province apply botanicals and associated indigenous knowledge to manage pests and diseases affecting their crops. The participants utilise 23 plants from 16 families for crop protection. The popularity of *C*. *annuum*, *A*. *cepa*, *D. cinerea*, *T*. *violacea* and *E*. *diversicolor* for crop protection was evident among the participants. Furthermore, *T*. *violacea*, *A*. *cepa*, *C*. *annuum*, *S*. *campylacanthum* and *P*. *pinaster* were identified as plants with multi-applications in the management of diverse pests and diseases. The highest Fic of 0.64 suggests that participants agreed on the utilisation of several plants for the management of fungal diseases affecting their crops. There was limited consensus or shared knowledge among smallholder farmers regarding the plants used to manage pests (vertebrate and invertebrate) and bacterial diseases affecting their crops. Taken together, the generated inventory of plants and associated indigenous knowledge is an indication of the awareness and the drive towards green-based approach as well as the dependence on natural resources for crop protection among smallholder farmers. These findings also contribute towards the global effort on the active documentation of the uses for valuable biodiversity to ensure their sustainability and conservation. Furthermore, these results provide baseline information for selecting and assessing the biological efficacy and phytochemical profiles of plants with potential for crop protection.

### Supplementary Information

**Additional file 1**. Supplementary Tables S1 & S2.

## Data Availability

We have included all datasets generated during the current study in the manuscript.

## Abbreviations

FC: Frequency of citations
Fic: Informant consensus factor
FNASREC: Faculty of Natural and Agricultural Sciences Research Ethics Committee
GC–MS: Gas chromatography mass spectroscopy
Nt: Number of taxa
Nur: Number of use report
RFC: Relative frequency of citation
UN SDGs: United Nations Sustainable Development Goals
UV: Use-value
