# Supplementary material for: Ethnobotanical insights on the management of plant pests and diseases by smallholder farmers in Mpumalanga Province of South Africa
Source: J Ethnobiol Ethnomed. 2024 Jul 31;20:71. doi: 10.1186/s13002-024-00711-x (PMC11293110; doi:10.1186/s13002-024-00711-x)
Supplement: Supplementary file 1 — Additional file 1. Supplementary Tables S1 & S2. [file 13002_2024_711_MOESM1_ESM.docx]

**SUPPLEMENTARY DATA**

**Supplementary Table S1.** Ecological overview and attributes of the population in the eight study sites. *Ethnic group, ^#^Language, ^$^Religion, ^+^Number of inhabitants was derived from ‘The Statistics South Africa’’ (Stats SA. Population and housing census indicators. 2011.); ^Ecology was derived from Mpumalanga Biodiversity Sector Plan Handbook (MTPA, 2014).

| Study site | Brooklyn | Chochocho | Drikoppies | Hlau hlau | Moloro | Origstad dam | Phakane | Tintswalo village |
| --- | --- | --- | --- | --- | --- | --- | --- | --- |
| Global Positioning System – GPS | 24.3625 S, 30.5851 E | 24.7014 S, 31.1169 E | 25.6991 S, 31.5638 E | 25.3566 S, 31.1764 E | 24.6078 S, 30.9787 E | 24.95357 S, 30.62978 E | 25.3708 S, 31.1912 E | (24.3435.1408 S, 31.429.9064 E) |
| Altitude (m) | 684 | 571 | 332 | 611 | 708 | 668 | 601 | 659 |
| *Ethnic group | Black African | Black African | Black African | Black African | Black African | Black African | Black African | Black African |
| ^Ecology | Vegetation type: Savannah | Vegetation type: Savannah | Vegetation type: Grassland and savannah | Vegetation type: Grassland and savannah | Vegetation type: Savannah | Vegetation type: Grassland and forest. | Vegetation type: Grassland and savannah | Vegetation type: Ssavannah |
|  | Climate: Warm  to hot, moist to wet summers and dry, mild to cool winters. | Climate: Warm  to hot, moist to wet summers and dry, mild to cool winters. | Climate: Warm  to hot, moist to wet summers and dry, mild to cool winters. | Climate: Warm  to hot, moist to wet summers and dry, mild to cool winters. | Climate: Warm  to hot, moist to wet summers and dry, mild to cool winters. | Climate: Warm  to hot, moist to wet summers and dry, mild to cool winters. | Climate: Warm  to hot, moist to wet summers and dry, mild to cool winters. | Climate: Warm  to hot, moist to wet summers and dry, mild to cool winters. |
|  | Hottest month: February (23 °C avg)  Coldest month:  June (16 °C avg)  Wettest month: December (135.6 mm avg | Hottest month: February (23 °C avg)  Coldest month:  June (16 °C avg)  Wettest month: December (135.6 mm avg) | Hottest Month:  February (23 °C avg)  Coldest Month:  June (16 °C avg)  Wettest Month:  December (135.6 mm avg) | Hottest Month:  February (23 °C avg)  Coldest Month:  June (16 °C avg)  Wettest Month:  December (135.6 mm avg) | Hottest month: February (23 °C avg)  Coldest month: June (16 °C avg)  Wettest month: December (135.6 mm avg) | Hottest month: February (23 °C avg)  Coldest month: June (16 °C avg)  Wettest month: December (135.6 mm avg) | Hottest Month:  February (23 °C avg)  Coldest Month:  June (16 °C avg)  Wettest Month:  December (135.6 mm avg) | Hottest month: February (23 °C avg)  Coldest month:  June (16 °C avg)  Wettest month: December (135.6 mm avg) |
|  | Windiest month: July (16 km/h avg) | Windiest month: July (16 km/h avg) | Windiest Month  July (16 km/h avg) | Windiest Month  July (16 km/h avg) | Windiest month: July (16 km/h avg) | Windiest month: July (16 km/h avg) | Windiest Month  July (16 km/h avg) | Windiest month: July (16 km/h avg) |
|  | Annual precipitation: 670.4 mm (per year) | Annual precipitation: 670.4 mm (per year) | Annual precipitation:  670.4 mm (per year) | Annual precipitation:  670.4 mm (per year) | Annual precipitation: 670.4 mm (per year) | Annual precipitation:670.4 mm (per year) | Annual precipitation:  670.4 mm (per year) | Annual precipitation: 670.4 mm (per year) |
| ^#^Language | Sepedi | Sepedi, Xitsonga, Sesotho | SiSwati, Xitsonga,  isiZulu, isiNdebele | SiSwati,  Xitsonga | Sepedi, Xitsonga, Sesotho | Sepedi | siSwati | Sepedi, Xitsonga, Sesotho |
| ^$^Religion | Christianity | Christianity | Christianity | Christianity | Christianity | Christianity | Christianity | Christianity |
| ^+^Number of inhabitants | 2,949 | 3,401 | 18,498 | 9,590 | 2,053 | 863 | 3,086 | 89 |

REFERENCES

MTPA. 2014. Mpumalanga Biodiversity Sector Plan Handbook. compiled by Lötter M.C., Cadman, M.J. and Lechmere-Oertel R.G.

Mpumalanga Tourism & Parks Agency, Mbombela (Nelspruit), Mpumalanga Province, South Africa.

Stats SA. Population and housing census indicators. 2011

**Supplementary Table S2.** The reported crops cultivated in the eight study sites. The botanical names were confirmed and verified using the ‘World Flora Online” (<http://www.worldfloraonline.org/>) and ‘Plants of the World Online’ (<http://www.plantsoftheworldonline.org/>). *Local/common name: S, Sepedi; Tso, Xitsonga; Swa, siSwati; Eng, English. ^#^FC = frequency of citation.

| Crop category | Local/common name | Scientific name and Family | Growth form | Biogeography status | ^#^FC (%) |
| --- | --- | --- | --- | --- | --- |
| Forage | Ummbila (Swa); Maize (Eng); Mahea (S); Mavele (Tso) | *Zea mays* subsp. *mays* (Poaceae) | Herb | Naturalised | 100 |
| Fruit | Mophopho (S); Papaya (Eng) | *Carica papaya* L. (Caricaceae) | Tree | Naturalised | 42 |
|  | Kalavatla (Tso); Emahawabhu (Swa); Legapu (S); Watermelon (Eng) | *Citrullus lanatus* (Thunb.) Matsum. & Nakai (Cucurbitaceae) | Herb | Naturalised | 4 |
|  | Apola (S); Apple (Eng), Apula (Tso); Emahabhula (Swa) | *Malus domestica*  (Suckow) Borkh. (Rosaceae) | Tree | Naturalised | 1 |
|  | Mango (Eng); Menku (S); Mongose (Tso) | *Mangifera indica* L. (Anacardiaceae) | Tree | Naturalised | 100 |
|  | Avocado (Eng); Abokado (S); Kotapeni (Swa) | *Persea americana* Mill. (Lauraceae) | Tree | Naturalised | 42 |
|  | Difaola (S); Ti prunes (Tso); I-prunes (Swa); Prunes (Eng) | *Prunus domestica* L. (Rosaceae) | Tree | Naturalised | 8 |
|  | Ematamatisi (Swa); Tamati (S); Tomato (Eng); Matamatisi (Tso) | *Solanum lycopersicum* L. (Solanaceae) | Shrub | Naturalised | 100 |
| Oil | Green beans (Eng); Ti beans (Tso); Dinawa (S); Emabhontisi (Swa) | *Phaseolus* *vulgaris* L.  (Fabaceae) | Herb | Naturalised | 14 |
|  | Bambara groundnuts (Eng); Dintlu (S); Emantongomane aseBambara (Swa) | *Vigna subterranea* (L.) Verdc. (Fabaceae) | Herb | Naturalised | 33 |
|  | Cowpea peas (Eng); Tinyawa (Tso); Dierekisi (S); Tindlubu (Swa) | *Vigna unguiculata* (L.) Walp. (Fabaceae) | Herb | Naturalised | 33 |
| Tuber | Amadumbe (Swa); Marope (S) | *Colocasia esculenta* (L.) Schott (Araceae) | Herb | Naturalised | 25 |
|  | Cassava (Eng.); Morogo wa Motombhula (S); Ntsumbula (Tso) | *Manihot esculenta* Crantz (Euphorbiaceae) | Shrub | Naturalised | 25 |
| Vegetable | Eie (S); Onion (Eng); Anyanisi (Swa); Nyala (Tso) | *Allium cepa* L. (Amaryllidaceae) | Herb | Naturalised | 100 |
|  | Red amaranths (Eng); Imbuya (Swa); Thyeke (Tso); Theepe (S) | *Amaranthus cruentus* L. (Amaranthaceae) | Herb | Naturalised | 75 |
|  | Dipete (S); Beetroot (Eng) | *Beta vulgaris* L. (Amaranthaceae) | Herb | Naturalised | 29 |
|  | Black jack (Eng); Moshitja (S) | *Bidens pilosa* L. (Asteraceae) | Herb | Naturalised | 8 |
|  | Cauliflower (Eng); Kholifolawa (S); I-Cauliflower (Swa) | *Brassica oleracea* var. *Botrytis* (Brassicaceae) | Herb | Naturalised | 1 |
|  | Khabetšhe (S); Kavichi (Tso); Cabbage (Eng); Emakhabishi (Swa) | *Brassica oleracea* var. *capitata* L. (Brassicaceae) | Herb | Naturalised | 17 |
|  | Pherefere (S); Chilli pepper (Eng); Epelepele; Virivirri (Tso) | *Capsicum annuum* L. (Solanaceae) | Herb | Naturalised | 45 |
|  | Epelepele (Swa); Pherefere (S); Chillies (Eng); Virivirii (Tso) | *Capsicum frutescens* L. (Solanaceae) | Herb | Naturalised | 94 |
|  | Chaya spinach (E); Sepeneše sa chaya (S); Xipinichi xa chaya (Tso); Sipinachi se chaya (Swa) | *Cnidoscolus aconitifolius* (Mill.) I.M.Johnst. (Euphorbiaceae) | Shrub | Naturalised | 12 |
|  | Jew’s mallow (Eng); Thelele (S); Ligusha (Swa); Guxe (Tso) | *Corchorus olitorius* L. (Malvaceae) | Herb | Indigenous | 83 |
|  | Pumpkin leaves (Eng); Moroho wa makwembe (Tso); Morogo wa mafodi (S) | *Cucurbita maxima* Duchesne (Cucurbitaceae) | Climber | Naturalised | 28 |
|  | Ticadzi (S); Digwere (S); Carrots (Eng) | *Daucus carota* L. (Apiaceae) | Herb | Naturalised | 14 |
|  | Lettuce (Eng); Letšhisi (S) | *Lactuca sativa* L. (Asteraceae) | Herb | Naturalised | 2 |
|  | Potato (Eng); Tapola (S); Mazambala (Tso); Lizambane (Swa) | *Solanum tuberosum* L. (Solanaceae) | Herb | Naturalised | 27 |
|  | Sepeneše (S); Xipinichi (Tso); Spinach (Eng); Sipinachi (Swa) | *Spinacia oleracea* L. (Amaranthaceae) | Herb | Naturalised | 75 |
